# Interferon at the crossroads of SARS-CoV-2 infection and COVID-19 disease

**DOI:** 10.1016/j.jbc.2023.104960

**Published:** 2023-06-24

**Authors:** Charles E. Samuel

**Affiliations:** Department of Molecular, Cellular and Developmental Biology, University of California, Santa Barbara, California, USA

**Keywords:** interferon, innate immunity, coronavirus, viral immunology, toll-like receptor, RIG-like receptor

## Abstract

A novel coronavirus now known as SARS-CoV-2 emerged in late 2019, possibly following a zoonotic crossover from a coronavirus present in bats. This virus was identified as the pathogen responsible for the severe respiratory disease, coronavirus disease-19 (COVID-19), which as of May 2023, has killed an estimated 6.9 million people globally according to the World Health Organization. The interferon (IFN) response, a cornerstone of antiviral innate immunity, plays a key role in determining the outcome of infection by SARS-CoV-2. This review considers evidence that SARS-CoV-2 infection leads to IFN production; that virus replication is sensitive to IFN antiviral action; molecular mechanisms by which the SARS-CoV-2 virus antagonizes IFN action; and how genetic variability of SARS-CoV-2 and the human host affects the IFN response at the level of IFN production or action or both. Taken together, the current understanding suggests that deficiency of an effective IFN response is an important determinant underlying some cases of critical COVID-19 disease and that IFNλ and IFNα/β have potential as therapeutics for the treatment of SARS-CoV-2 infection.

The emergence of a novel coronavirus from the Wuhan area of the Peoples's Republic of China in late 2019, a virus initially known as nCoV (1) but since designated severe acute respiratory syndrome coronavirus 2 (SARS-CoV-2), was determined to be the causative agent of a coronavirus pneumonia-like disease 2019 (2, 3, 4, 5). This new coronavirus of probable bat origin infected humans and rapidly triggered a global pandemic (6, 7, 8).

SARS-CoV-2 has the capacity to cause severe respiratory illness. The virus spread quickly, and its fitness continued to evolve (9, 10, 11). As of May 2023, there were ∼6.9 million deaths and 767 million confirmed cases worldwide reported to the World Health Organization (WHO), with more than one million deaths and 103 million confirmed cases in the United States alone from the pandemic (12). The disease caused by SARS-CoV-2 came to be known as COVID-19. The virus responsible for the COVID-19 pandemic was established early during 2020 as a betacoronavirus, and in a demonstration of the power of science and technology, the ∼30-kb nucleotide sequence of the viral genome was quickly elucidated and that knowledge set the stage for diagnostic and vaccine development (13, 14). The nCoV sequence revealed high genetic similarity with the SARS-CoV betacoronavirus responsible for the earlier SARS epidemic from 2002 to 2003. The new virus was designated SARS-CoV-2 because, by taxonomic criteria, it is not a different viral species than SARS-CoV. The SARS-CoV and SARS-CoV-2 sequences show ∼87% similarity. A bat coronavirus, RaTG13, has ∼96% sequence identity with SARS-CoV-2 and is its nearest known relative (7, 13, 14).

During the course of the pandemic, it was realized that some individuals infected by SARS-CoV-2 remained asymptomatic for disease or presented with mild illness, whereas others developed severe respiratory disease and pneumonia and did not survive. Why the range of outcomes? No doubt there are multiple reasons. Among them emerged an understanding of the importance of the interferon response, a cornerstone of innate immunity, in limiting disease severity. Focusing on studies that probe the role of the interferon system in determining the outcome of SARS-CoV-2 coronavirus infection, this review assesses biological responses and molecular mechanisms underpinning the interferon response and considers evidence that some of the severe COVID-19 cases are associated in part with a dysregulated interferon (IFN) response. We consider questions of the sensitivity of SARS-CoV-2 replication to the antiviral actions of different types of IFNs in culture and *in vivo*; the IFN-inducing capacity of SARS-CoV-2; molecular mechanisms by which the SARS-CoV-2 virus is implicated to antagonize IFN responses; and how genetic variability of the SARS-CoV-2 virus and the human host play roles in determining the overall robustness of IFN antiviral actions.

## The SARS-CoV-2 virus

The novel coronavirus SARS-CoV-2 emerged during late 2019 (1, 2). This virus was identified quickly as the pathogen responsible for a severe respiratory disease (3, 8) designated coronavirus disease-19 or COVID-19 that spread rapidly to become a pandemic. Interferon is implicated to play a key role in determining the outcome of infection by the SARS-CoV-2 coronavirus.

### SARS-CoV-2 is a betacoronavirus

As such, SARS-CoV-2 is classified as a member of the Order Nidovirales, Family Coronaviridae, and Genus Betacoronavirus (14, 15, 16, 17). The classification is based on the sequence. The sequences of the SARS-CoV-2 genome obtained from five patients early during the COVID outbreak were nearly identical and shared 79.6% sequence identity with the SARS-CoV sequence from the 2002 to 2003 epidemic and, furthermore, was closely related to several bat coronaviruses, suggesting a potential zoonotic transmission to human from bat, possibly involving intermediate hosts (7, 18). Viruses classified as betacoronaviruses include the murine coronavirus, mouse hepatitis virus (MHV), which has been a valuable experimental model; the endemic human coronaviruses HCoV-OC43 and HCoV-HKU1, which generally cause mild respiratory infections and the common cold but sometimes can be more severe in the very young and the elderly; and, three additional highly pathogenic viruses that all caused epidemics or the current pandemic. These are the severe acute respiratory syndrome coronavirus (SARS-CoV) that emerged in 2002 in China; the Middle East respiratory syndrome-related coronavirus (MERS-CoV) that emerged in 2012 in Saudi Arabia; and now, the severe acute respiratory syndrome coronavirus 2 (SARS-CoV-2) that emerged in 2019 in China (1, 2, 18, 19, 20). The classification of these viruses as betacoronaviruses is based on phylogenetic clustering and pairwise distances from the comparison of sequences of key regions of the replicase-transcriptase polyprotein that taxonomically define coronavirus identity (2, 15).

### Genome

The SARS-CoV-2 genome is single-stranded, positive-sense RNA (+ssRNA) of 29,870 nucleotides, excluding the polyadenylated tail (13); Genbank reference sequence NC_045512. The full-length genome is associated with viral nucleocapsid N protein and encapsidated within an enveloped virion of ∼80 to 90 nm diameter. Genome RNA possesses a 7-methyl-G 5′-cap structure and a 3′-polyadenylate tail and encodes 4 virion structural proteins within the 3’ ∼third of the genome: Spike (S); Envelope (E); Membrane (M); and Nucleocapsid (N). Sixteen nonstructural proteins (nsp 1–16) are encoded within the 5’ ∼two-thirds of the genome. These are essential for virus replication, for example, by providing the machinery required for viral RNA replication and modification. Several accessory gene ORF products non-essential for replication are encoded within the 3′-genome region. SARS-CoV-2 and SARS-CoV, in contrast to some other coronaviruses, do not possess a viral 2′-5′ phosphodiesterase gene, such as is found in MHV, that impairs the 2,5A innate IFN response, which will be discussed later.

### Multiplication cycle

**Figure 1:**
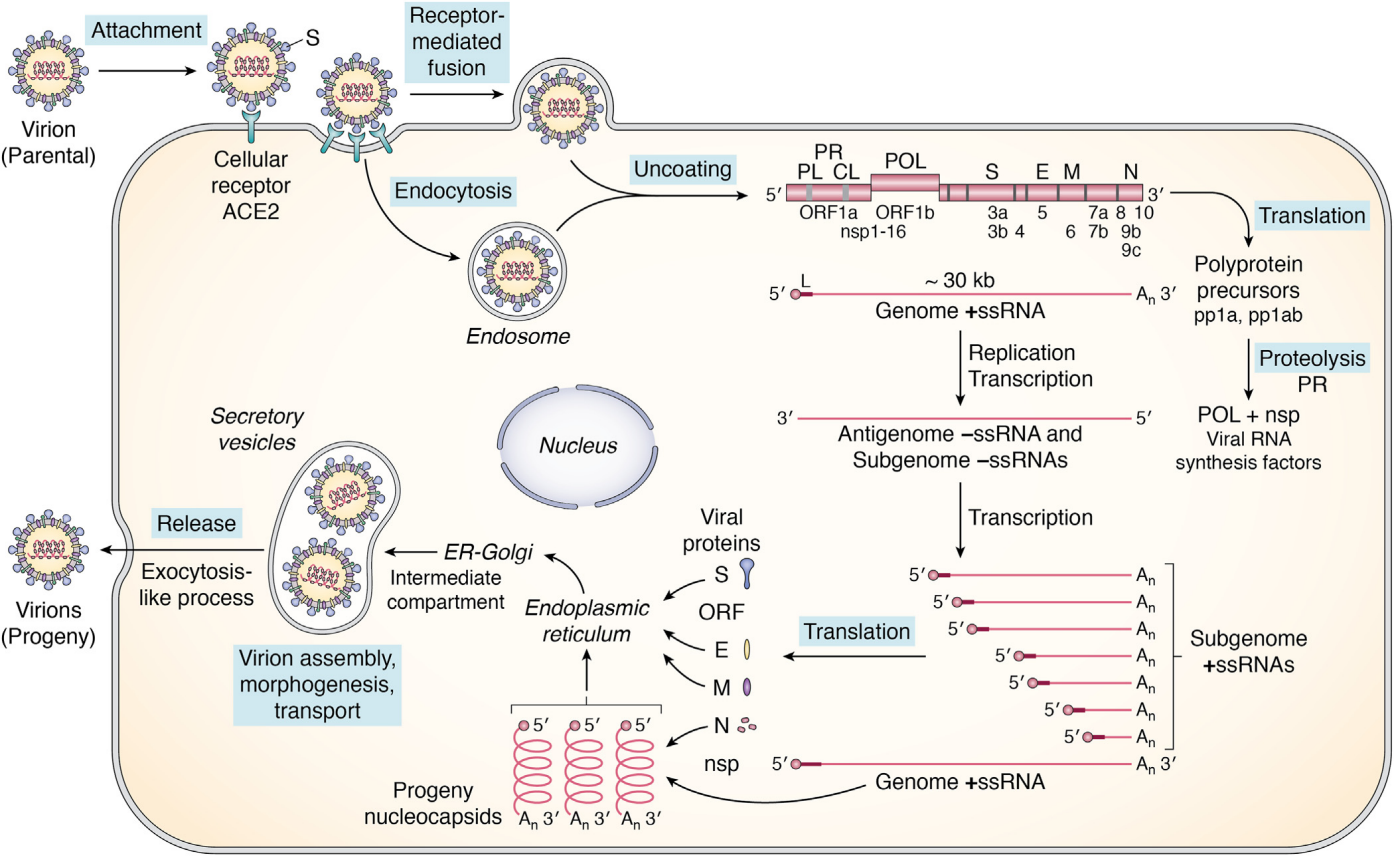
**Schematic diagram summary of the SARS-CoV-2 coronavirus multiplication cycle**. SARS-CoV-2 virus possesses a positive-sense, single-stranded RNA genome of ∼30-kb. Virus multiplication occurs in the cytoplasm. Following virion attachment by binding of the viral S spike protein to the cellular ACE2 receptor protein, entry occurs by receptor-mediated fusion of the viral envelope with the host plasma membrane, releasing the ribonucleocapsid complex containing genome +ssRNA into the cytoplasm. The 5′-capped genome RNA functions as mRNA and is translated directly by the host cell protein-synthesizing machinery to generate two polyprotein precursor products, pp1a from ORF1a, and pp1ab from ORF1a and ORF1b following a −1 ribosomal frameshift to read frame1b. The polyprotein precursors undergo autoproteolytic processing by viral PL and CL proteases to generate nonstructural proteins (nsp 1–16) that include the viral RNA polymerase and factors required for viral RNA biogenesis. The parental genome +ssRNA next functions as a template for replication and transcription to produce full-length antigenomic –ssRNA (that is the template for synthesis of full length +ssRNA progeny genomes) and a set of subgenomic –ssRNAs (that are the templates for subgenomic +ssRNAs that are the mRNAs for synthesis of virion structural proteins N, M, E and S as well as several ORF accessory proteins that affect the host response to infection). Viral RNA synthesis occurs in association with cellular membrane structures, as does progeny virion assembly and morphogenesis followed by release by an exocytosis-like process.

The multiplication cycle of the SARS-CoV-2 virus is summarized in Figure 1. The molecular virology and biology of the coronavirus life cycle have recently been reviewed (15, 16, 17). Initiation of SARS-CoV-2 infection involves virion attachment by the viral spike protein to the major host cell receptor, angiotensin-converting enzyme II (ACE2), a protein present on the surface of many types of cells in the respiratory tract. Virion penetration into the host cell occurs by receptor–mediated endocytosis or by direct fusion between the virion envelope and the host cytoplasmic membrane, mediated by the host cell TMPRSS2 serine protease-activated viral spike S2 fusion domain. Then virion uncoating occurs within the infected cell to release the nucleocapsid containing the ∼30-kb +ssRNA genome (14, 16) to the cytoplasm. Replication occurs within the cytoplasm. The viral parental genome +ssRNA first functions as mRNA and is translated directly by the host protein-synthesizing machinery to generate two polyprotein products, polyprotein pp1a and polyprotein pp1ab. The larger pp1ab protein is synthesized following a programmed translational minus 1 ribosomal frameshift. The pp1a and pp1ab proteins undergo autoproteolytic cleavages to generate mature nsp 1 to 16 protein products that fulfill key functions during virus replication (13, 15, 18, 21, 22). Among the nsp are two viral proteases, the papain-like protease (PLpro, a domain contained within nsp3) and the chymotrypsin-like protease (CLpro, nsp5) that generate by proteolytic processing the nsp necessary for viral RNA replication and transcription, including the RNA polymerase (nsp12), cofactors acting with the polymerase complex (nsp7, 8), a helicase (nsp13), 5′-G capping and N7- and 2′O-methylation activities (nsp9, 10, 14, 16), two ribonucleases, a 3′-5′ exonuclease (nsp14) and a U-selective endoribonuclease (nsp15), and proteins involved in membrane remodeling for RNA synthesis (nsp3, 4, 6). The parental genome +ssRNA, in addition to functioning as mRNA, also functions as the template for replication and transcription to produce full-length antigenome −ssRNA, the template for full-length progeny RNA genomes, as well as a set of subgenomic −ssRNAs. The subgenomic -ssRNAs then serve as the templates for transcription to yield the subgenomic +ssRNAs that are the mRNAs for the virion structural proteins S, E, M, and N, and the accessory ORF products. The +ssRNA progeny genome molecules assemble with N protein to form progeny helical nucleocapsids. Virion assembly and morphogenesis occur through budding at an intracellular membrane, the ER-Golgi intermediate compartment (ERGIC), yielding progeny virions within secretory vesicles that are subsequently released by an exocytosis-like process (Fig. 1).

## The interferon system

**Figure 2:**
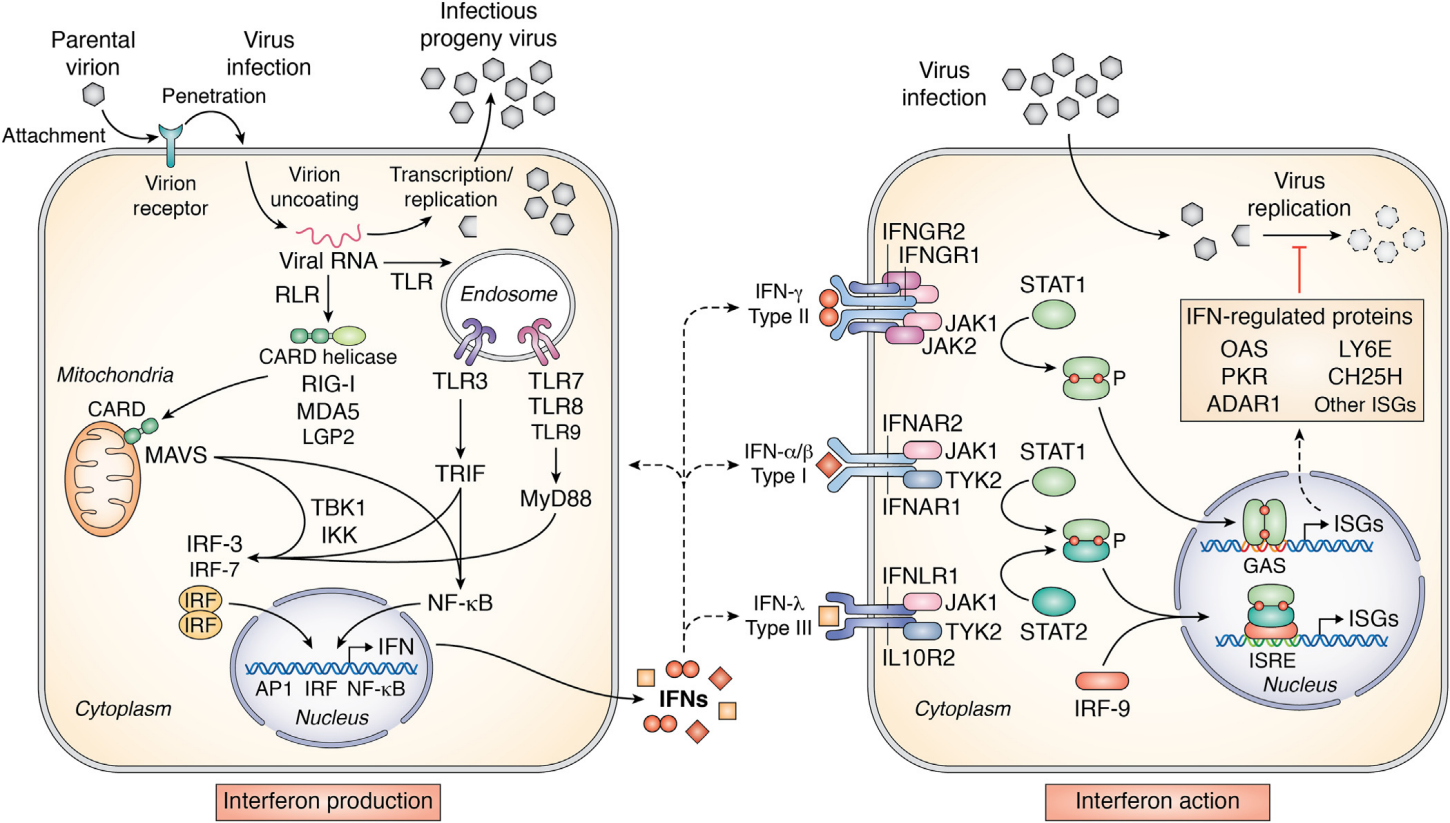
**Schematic diagram summarizing the interferon system response activated by virus infection leading to interferon production (*left*) and interferon action (*right*).** The IFN-producing cell shown on the left illustrates a cell induced to synthesize IFN in response to virus infection. The cytoplasmic RLR MDA5 and endosomal TLRs sense viral (nonself) and possibly cellular (self) nucleic acids and signal *via* the mitochondrial adaptor MAVS and the TRIF and MyD88 adaptors, respectively, to activate the interferon regulatory (IRF) 3 and 7 and NF-κB transcription factors to transcriptionally activate IFN expression. The IFN-treated cell shown on the right depicts a cell induced to express IFN-regulated proteins by JAK-STAT signal transduction leading to transcriptional activation of interferon-stimulated gene (ISG) expression. ISG expression in response to type I α/β/ω or type III λ IFN treatment occurs *via* ISRE element regulation, and type II γ IFN treatment *via* GAS element regulation. Among the ISGs implicated to play a role in the antiviral action of IFNs against SARS-CoV-2 virus are OAS1 2′5′-oligoadenylate synthetase, PKR protein kinase, LY6E lymphocyte antigen 6 and CH25H cholesterol 25-hydroxylase.

Interferons were discovered based on their antiviral activity (23, 24). During studies of viral interference, Isaacs and Lindenmann observed that influenza virus-infected chick cells produced a secreted, soluble factor that possessed antiviral activity against both the homologous inducing influenza virus but also against heterologous viruses (23). Similar findings were described shortly thereafter by Nagano and Kojima (24). We now understand the interferon (IFN) system in considerable biochemical detail (25, 26, 27, 28, 29, 30, 31, 32, 33). Figure 2 summarizes interferon signal transduction pathways that trigger IFN production (*left*) and IFN action (*right*) in virus-infected cells.

IFNs are a multigene family of inducible cytokines (25, 26, 27, 33). They are classified into three types: type I IFN, also known historically as viral IFN, of which there are several members, including multiple subtypes of α and a single β and ω; type II IFN, also known as immune IFN, of which there is a single member (γ); and, the most recently identified type III IFN (λ), of which there are multiple subtypes. Type I IFNs together with the type III IFNs are the major antiviral IFNs. IFNs are the cornerstone of the innate immune response. Transcriptional activation of IFN gene expression is triggered by multiple different nucleic acid sensors in virus-infected cells, sensors including the cytosolic RIG-I-like receptors (RLRs) and endosomal Toll-like receptors (TLRs) (28, 29). RLRs act through the signaling adaptor protein MAVS—also known as IPS-1—and TLRs through adaptors TRIF and MyD88, that lead to activation of latent cytoplasmic transcription factors, interferon regulatory factors IRF3 and IRF7 and NFκB *via* IKK, TBK, and TRAF proteins (Fig. 2, *left*).

While SARS-CoV-2 infection triggers the MDA5 dsRNA sensor to induce IFN expression (34, 35, 36, 37), the precise nature of the viral dsRNA that activates MDA5 and also additional dsRNA sensors including the dsRNA-dependent protein kinase (PKR) and dsRNA-dependent 2′-5′ oligoadenylate synthetases (OAS) (30, 38) is not yet known. It is tempting to speculate that the activator viral dsRNA effector derives from SARS-CoV-2 viral replication intermediate structures, but this is not yet established and regions within viral ssRNAs with secondary structure cannot be excluded as fulfilling an innate activator function. Indeed, in the case of OAS, it has been shown that OAS1 binds to SARS-CoV-2 viral RNAs in a remarkably specific manner with two conserved stem-loops in the SARS-CoV-2 5′-UTR constituting the principal viral target (39). Conceivably this RNA region likewise possesses sufficient ds-character to activate PKR as well. SARS-CoV-2 structure and replication have been characterized by Cryo-EM (40). RNA filaments of a diameter consistent with dsRNA are described inside double-membrane vesicles associated with viral replication (40). Whether this RNA would be accessible to dsRNA sensors such as MDA5, PKR, and OAS1 is not well understood. TLR7 and TLR8 also are implicated as key sensors of SARS-CoV-2 ssRNAs (41, 42). A systematic screen for SARS-CoV-2 genome-derived ssRNA revealed viral RNA fragments that activate TLR7 and TLR8 and induce cytokine release from macrophages and microglia (41). And, *TLR7* variants have been identified in patients with critical COVID-19 pneumonia (42, 43).

IFNs exert their antiviral actions by binding to type-specific cognate cell surface receptors that then trigger transcriptional activation and expression of IFN-stimulated genes (ISGs) by JAK-STAT signaling (Fig. 2, *right*). IFNs may act in either an autocrine or paracrine manner. Most kinds of cells express type I IFN receptors, whereas type III IFN receptors are primarily expressed on epithelial cells and type II IFN receptors on many cell types including cells of the immune system. IFN binding activates receptor-associated *Janus* family tyrosine kinases (JAK1, JAK2, and TYK2) that leads to the activation of latent cytoplasmic STAT (signal transducer and activator of transcription) factors. IFN-type specificity of signaling is achieved through the use of high-affinity receptors present on different kinds of cells, the use of the receptor-associated JAK kinases in overlapping pairs by the different types of IFN, and the use of different combinations of STAT factors to activate ISG transcription (27, 31, 33, 44, 45). For type II IFNγ, JAK1 and JAK2 activation lead to STAT1 homodimerization and nuclear translocation of the gamma IFN-activated GAF factor that binds the cis-acting GAS DNA element to drive ISG expression by IFNγ. For types I and III IFNs, following the binding of these IFNs to their cognate receptors, JAK1 and TYK2 lead to activation of STAT1 and STAT2 that heterodimerize and, together with IRF9, form IFN-stimulated gene factor ISGF3. Upon nuclear translocation, ISGF3 binds at cis-acting ISRE DNA elements to drive the ISG expression induced by α, β, ω, and λ IFNs (Fig. 2, *right*). Multi-species comparison of interferon responses to type I IFN reveals a conserved core of 62 ISGs (46). Some ISGs display antiviral activity that is surprisingly selective, but not all ISGs are antiviral or are selective (25, 32, 33); adenosine deaminase acting on RNA1, for example, is an ISG that is often proviral (38).

## Induction of interferon by SARS-CoV-2 virus

The ability of SARS-CoV-2 infection to induce IFN appears to vary, dependent upon the conditions of infection and disease (34, 47, 48, 49, 50, 51, 52, 53). The IFN response, however, is generally delayed during infection with the β-coronaviruses MHV and SARS-CoV-2 compared to other viruses. SARS-CoV-2 infects and replicates in a variety of cell types, including primary human airway epithelial (pHAE) cell cultures, with progeny virion release more efficient on the apical compared to the basolateral surface of infected cells. While infected cells display a transcription signature of upregulated proinflammatory cytokine expression, including IL-6, IL-8, TNF-α, and CXCL8, induction of types I and III IFNs can be variable and poor (48, 49, 54). An impaired type I IFN response is seen in severe COVID-19 patients (50). Additionally, young infants exhibit robust functional antibody responses but restrained IFNγ production following SARS-CoV-2 infection compared to adults (51).

Screening of nucleic acid sensors implicated in the sensing of infection by various RNA viruses revealed that MDA5, a RIG-like receptor, is the major sensor that detected SARS-CoV-2 infection to activate an IFN production response, albeit delayed (34, 35, 36, 37, 55). Messenger RNA and protein levels of IFN types I and III were significantly elevated in infected Calu3 human lung epithelial cells compared to uninfected cells. SARS-CoV-2 also induced an IFN response in iPSC-derived airway epithelial cells. Single-cell transcriptomic analyses of SARS-CoV-2-infected human airway bronchial epithelial cells identified ciliated cells as a major target at the onset of infection, with a tropism that then expanded to other epithelial cell types, including basal cells (54, 56). Expression of IFN β and λ transcripts was observed in cells co-expressing SARS-CoV-2 viral transcripts; interferon-stimulated gene (ISG) expression was observed both in infected and in bystander cells, consistent with both autocrine and paracrine signaling following SARS-CoV-2 infection (56). SARS-CoV-2 evades IFN activation in respiratory epithelial cells, resulting in a delayed response in bystander cells (57). A screen of 75 microbial ligands that activate diverse signaling pathways identified cyclic dinucleotides, agonists of STING (endogenous stimulator of interferon genes) as antiviral. The STING agonist diABZI inhibited SARS-CoV-2 infection by transiently stimulating IFN signaling; diABZI restricted viral replication in primary human bronchial epithelial cells and in mice (57).

Triggering of type I IFN production by the MDA5 sensor following infection by SARS-CoV-2 is dependent upon ISG15 conjugation. ISGylation of MDA5 promotes its oligomerization and thereby triggers activation of innate immunity against a range of viruses, including coronaviruses; the ISG15-dependent activation of MDA5 is antagonized by direct de-ISGylation triggered by PLpro of SARS-CoV-2 (55). Interestingly, in the context of potential intermediate hosts involved in the zoonotic transmission of SARS-CoV-2, pangolins reportedly lack the MDA5 RNA sensor that initiates the innate immune response to coronavirus infection (37).

Kinetic analyses revealed that the increases in IFN β and λ expression were delayed in SARS-CoV-2 infected cells (30, 47, 48, 56, 57, 58, 59), and while SARS-CoV-2 triggers an MDA5-dependent IFN response, the IFN produced was unable to control viral replication in lung epithelial cells (36). The effect of SARS-CoV-2 infection of placenta-derived human trophoblast stem cells (TSC) showed that the virus was able to productively replicate in TSC-derived syncytiotrophoblast and extra-villous trophoblast cells but not in undifferentiated TSC. TSC-derived cells elicited an IFN-mediated response upon infection (60). In a single-cell RNA sequencing study of a primary cell model of human nasal epithelium cells infected with SARS-CoV-2, the host type I and III IFN responses were delayed relative to the onset of viral gene expression, as compared with other respiratory viruses (59).

## Interferons can inhibit SARS-CoV-2 replication

SARS-CoV-2 virus replication is inhibited by type I IFN α and β, type II IFN γ and type III IFN λ in cell culture (49, 57, 58, 59, 61, 62). However, the relative sensitivity depends both on the type of IFN and the cell line utilized to assess IFN antiviral activity. For quantitative comparison between studies of the sensitivity of SARS-CoV-2 virus multiplication to IFN antiviral action, it is necessary to know the type and subspecies of IFN utilized; the time and concentration of IFN treatment; the host cell line used for measurement of infectious virus yield reduction; and the conditions of the virus growth assay including multiplicity of infection and whether the assay was single cycle. In one of the initial comparative assessments of the antiviral activity of type I and type III IFNs against SARS-CoV-2 compared to SARS-CoV in epithelial cell lines (human Calu3, simian Vero E6), the results showed that both IFNs inhibited SARS-CoV-2. By contrast, SARS–CoV was inhibited only by type I IFN in these cell lines (58). SARS-CoV-2 generally exhibited a broader IFN sensitivity than SARS-CoV (58, 62). Treatment of cells with either type I β or type III λ IFN inhibited SARS-CoV-2 replication in a manner that correlated with induction of antiviral ISGs (49, 59). Susceptibility to IFN treatment also differed between SARS-CoV-2 and SARS-CoV in subsequent studies, with SARS-CoV-2 more sensitive than SARS-CoV to type I IFN, either α or β, in Vero and Calu3 cells (58, 62, 63, 64). Blocking of JAK-STAT signaling with ruxolitnib enhanced SARS-CoV-2 replication in IFN-induction-competent Calu3 cells, whereas in Vero cells, which are IFN-induction-deficient, there was no discernible effect of drug treatment on virus replication (58). Sensitivity of the SARS-CoV-2 and SARS-CoV to inhibitors of coronavirus replication (remdesivir, alisporivir) was similar, although SARS-CoV-2 infection was substantially more sensitive to pre-treatment with IFN (63).

A direct comparison of the potency of 17 different recombinant human type I IFNs (12 α, one β, one ω) and type III IFNs (three λ) against diverse SARS-CoV-2 variants in A549-ACE2 cells revealed that different IFNs inhibited SARS-CoV-2 to various degrees in cell culture (65). Overall, IFN α8, β, and ω were among the most potent, and λ IFNs were somewhat surprisingly the least potent against SARS-CoV-2. Furthermore, emerging SARS-CoV-2 variants were more resistant to the antiviral actions of IFNs than ancestral early pandemic isolates. Evidence for increasing IFN resistance of SARS-CoV-2 also was seen in primary human bronchial epithelial cells. Examination of ancestral SARS-CoV-2 and five variants of concern, including the B.1.1.7 (alpha), B.1.351 (beta), P.1 (gamma), B.1.617.2 (delta), and B.1.1.529 (omicron) lineages, suggested that relative to ancestral isolates, SARS-CoV-2 variants of concern exhibited increased IFN resistance (65). This possibly indicates that evasion of innate IFN immunity may be one of the driving forces for SARS-CoV-2 evolution. The mechanistic basis of the antiviral activity differences of different type I IFN subtypes is not well understood, although transcriptomic analysis of human airway epithelial cells has revealed different immune transcript signatures (66).

When SV40-transformed fibroblasts were derived from COVID disease patients with deficiencies in TLR3, IRF7, IFNAR1, TYK2, or STAT2, these cells were unable to control SARS-CoV-2 replication normally in cell culture compared to healthy donor cells used as positive controls (67, 68). Cells from TYK2- and STAT2-deficient patients also failed to induce ISG expression in response to either type I IFNα treatment or SARS-CoV-2 infection. These results obtained with patient-derived cells further suggest the importance of a type I IFN response in controlling virus infection and prevention of severe COVID pneumonia.

## IFN-stimulated genes that inhibit SARS-CoV-2 replication

The antiviral actions of IFNs are mediated by interferon-stimulated genes (ISGs) of which there are several, ∼60 core ISGs, that have been identified following type I IFN treatment (32, 46). Some ISGs have the capacity to individually inhibit the replication of some viruses, as demonstrated by overexpression and gene knockout studies. These ISGs include the dsRNA-activated PKR kinase and 2′-5′-oligoadenylate synthetase-RNase L responses that have been extensively characterized and represent key antiviral ISGs that inhibit a number of viruses (25, 30, 69). However, *in vivo* in an IFN-treated, virus-infected host, ISGs would have the potential to act in combination with each other. Indeed, screens have identified multiple ISGs that possess anti-SARS-CoV-2 activity.

### ISG screens

Screens of ISGs carried out using different strategies have identified ISGs that restrict SARS-CoV-2 replication (39, 70, 71, 72, 73). A gain-of-function screen of ISGs, initially by ectopic overexpression in 293T cells, revealed a subset of ∼65 ISGs that mediated the restriction of SARS-CoV-2 (71). These included endosomal factors inhibiting viral entry, RNA-binding proteins suppressing viral RNA synthesis, and endoplasmic reticulum (ER)/Golgi-resident ISGs inhibiting progeny virion assembly/egress at late stages of multiplication. In addition to several broadly acting antiviral ISGs, eight ISGs that specifically inhibited SARS-CoV-2 and SARS-CoV replication were identified. Among the ISGs identified by overexpression screening were LY6E (lymphocyte antigen 6 complex) (70, 71, 73), which inhibits virus entry, and BST2 (bone marrow stromal antigen2)/tetherin, which impairs virion release (71). Furthermore, BST activity was antagonized by the SARS-CoV-2 ORF7a protein (71).

The ability of potential ISGs to affect SARS-CoV-2 replication was assessed in a different study using a CRISPR/Cas9 screen designed to identify restriction factors of SARS-CoV-2 replication in human A549 epithelial lung cells (72). Core IFN pathway genes, including those encoding IFN action signaling components, the IFNAR1 receptor and STATs 1 and 2, were found as antiviral factors, providing a validation of the screening strategy. In addition, LY6E identified in a prior gain-of-function screen (70) and DAXX (death domain-associated protein 6), IFI6 (IFNα inducible protein 6), APOL6 (apolipoprotein 6), and HERC5 (HECT and RLD domain containing E3 ubiquitin protein ligase 5) were found (72).

### ISGs that inhibit an early step of SARS-CoV-2 replication

LY6E and CH25H (cholesterol 25-hydroxylase) are two ISGs that impair coronavirus initiation of infection by impairing fusion and entry (70, 71, 73). DAXX also acts at an early step to inhibit virus replication (72).

LY6E is a restriction factor that acts to restrict viral entry of SARS-CoV-2 as well as SARS-CoV and MERS-CoV (70). LY6E inhibits virion entry into the host cell by impairing the envelope spike-mediated membrane fusion during the initiation of infection. In a mouse model, knockout mice lacking LY6E in immune cells are highly susceptible to MHV, whereas constitutive expression of LY6E in the mouse is protective against MHV infection (70).

CH25H also is an ISG that acts to restrict viral entry (73, 74). By using an ISG screen against replication-competent vesicular stomatitis eGFP reporter viruses with either full-length SARS-CoV spike protein or the SARS-CoV-2 spike in place of the native VSV glycoprotein (G), CH25H and its enzymatic product 25-hydroxycholesterol (25HC) were identified as inhibitors of SARS-CoV-2 replication (73). CH25H inhibits SARS-CoV-2 and other coronaviruses by depleting membrane cholesterol. Cholesterol is converted to 25-hydrocholesterol (25HC) by the hydroxylase. 25HC blocks spike-mediated membrane fusion and, hence, virus entry through mobilizing accessible cholesterol from the plasma membrane. 25HC inhibits SARS-CoV-2 infection in lung epithelial cells and viral entry in human lung organoids. Mechanistically, 25HC inhibits viral membrane fusion by activating the ER-localized acyl-CoA:cholesterol acyltransferase (74).

DAXX is an ISG restriction factor that acts at an early, post-entry step of multiplication to inhibit the replication of both SARS-CoV-2 and SARS-CoV (72). DAXX is a scaffold protein present in PML nuclear bodies; SARS-CoV-2 infection triggers the relocalization of DAXX to the cytoplasm. SARS-CoV-2 virus has evolved a countermeasure to DAXX antiviral action; the viral PLpro promotes DAXX degradation. Basal expression of DAXX was sufficient to limit replication of SARS-CoV-2, though overexpression further limited infection. Knocking out DAXX only partially rescued SARS-CoV-2 replication in IFN-treated cells, consistent with the notion that other IFN effectors in addition to DAXX are likely also effective antiviral factors against SARS-CoV-2.

### dsRNA-dependent responses

Infection by SARS-CoV-2 activated dsRNA-dependent ISG responses in respiratory epithelial-derived cells, including two dsRNA-dependent antiviral pathways, the 2′,5′-oligoadenylate synthetase–RNase L response and the PKR protein kinase response, while generally inducing minimal levels of IFN (75). This is in contrast to MERS-CoV, which effectively inhibited IFN signaling and the OAS-RNase L and PKR dsRNA-dependent pathways (76). Activation of IFN or OAS-RNase L was not observed in induced pluripotent stem cell-derived alveolar type 2 cells (iAT2)--a major cell type infected in the lung-- and cardiomyocytes (iCM), whereas PKR activation was seen in iAT2 and iCM. In SARS-CoV-2–infected Calu3 and A549^ACE2^ lung-derived cell lines, IFN induction remained weak. However, activation of the OAS-RNase L and PKR responses was observed with SARS-CoV-2 (75). In contrast, with MERS-CoV, nsp15 endoU and the accessory proteins 4a (a dsRNA-binding protein) and 4b (a phosphodiesterase) act together to effectively suppress the dsRNA-mediated innate immune responses and enhance MERS-CoV replication (76).

ISG expression screening furthermore revealed that the dsRNA-dependent 2′-5′-oligoadenylate synthetase 1 (OAS1) inhibits SARS-CoV-2 (39). A common splice-acceptor single-nucleotide polymorphism (Rs10774671) governs whether patients express prenylated OAS1 isoforms that are membrane-associated and sense SARS-CoV-2 RNAs, or if they express a cytosolic nonprenylated OAS1 that does not efficiently sense SARS-CoV-2. In hospitalized patients, expression of prenylated OAS1 was associated with protection from severe COVID-19 disease, consistent with the notion that OAS1 represents a major component of a protective antiviral response (39).

Finally, the p150 isoform of ADAR1 adenosine deaminase acting on dsRNA is IFN inducible (38, 77) and has been implicated to play a role in SARS-CoV-2 infections (78, 79, 80). While reduced A-to-I editing of host cellular *Alu* sequences characteristic of an ADAR editing signature has been described in severe COVID-19 disease (78), the significance of such sequence changes in the SARS-CoV-2 transcriptome is not yet clear (79, 80). One consequence of A-to-I editing is destabilization of dsRNA structures that then suppresses both IFN production and action (38). Different characteristic features of viral dsRNA structures are sensed by the MDA5 compared to the RIG-I RLR sensor (81). ADAR1 clearly edits both viral and cellular RNAs to suppress the activation of the MDA5 sensor (38) as knockout of *Mda5* complements the embryonic lethality phenotype that is characteristic of the mouse *Adar1* and *Adar1* p150 knockouts (82, 83).

### IFITMs

IFN-induced transmembrane proteins (IFITMs) have been described to have both antiviral and proviral activities with respect to SARS-CoV-2. Counterintuitively, among the IFN-inducible ISGs, the IFITM family, especially IFITM2, is reportedly required for efficient replication of SARS-CoV-2 in cell culture (84, 85). For other viruses, IFITMs typically are antiviral, perhaps as anticipated for genes regulated by IFN, and two studies observed an inhibitory activity for them with the SARS-CoV-2 and MERS-CoV viruses (86, 87). Interestingly, expression of virion S protein alone triggers syncytia formation from fusion with neighboring ACE2-positive cells, and IFITM proteins inhibit the S-mediated fusion (88). The proviral enhancing effect of the IFITM2 transmembrane protein appears possibly to involve interactions between viral S protein and the N-terminal region of IFITM2 that promotes subsequent virus-cell fusion in early endosomes. Knockdown of IFITM2 in Calu3 cells reduced virus replication, whereas antibodies to IFITM2 protected against SARS-CoV-2 mediated cytopathic effect (85). Analysis of five variant SARS-CoV-2 viruses, alpha, beta, gamma, delta, and omicron, revealed that all five variants of concern maintained the dependency on IFITM2 protein for efficient virus replication (84). The reason for the conflicting findings between studies that showed proviral (84, 85) compared to antiviral (86, 87) effects of IFITM2 for replication of betacoronaviruses is unclear, but it may relate in part to differences in the details of assays utilized. IFITM3 knockout (KO) mice infected with SARS-CoV-2 show extreme weight loss and lethality compared to mild infection in WT mice. The KO mice have higher lung viral titers, suggesting that IFITM3—like IFITM2 in some studies—has an antiviral role *in vivo* with SARS-CoV-2 (89). Transcriptomic analysis of infected lungs shows upregulation of gene signatures associated with IFNs and inflammation.

## Antagonism of the interferon response by SARS-CoV-2 virus

The robustness of an IFN response can be modulated by either viral or cellular gene products that act, for example, to impair the IFN-triggered JAK-STAT signaling pathway or inhibit the activity of ISG products (25, 26, 38, 90, 91, 92, 93, 94). In cultured cells and in mouse models for SARS-CoV (95) and SARS-CoV-2 (96) infections, type I IFN signaling was required for ISG induction but such signaling was not necessarily sufficient to control virus replication. Historically, beginning with the identification of the poxvirus E3L protein (97) and the adenovirus VAI RNA (98), a number of different viral protein and RNA gene products were subsequently identified from multiple additional viruses that impair the IFN response, inhibiting either IFN production or IFN action (25, 93). Among these antagonists are several SARS-CoV-2 gene products observed as inhibitors of the IFN response (20, 21, 94, 99, 100, 101, 102).

Ectopic overexpression of viral gene products is one approach to assess their ability to antagonize the host IFN response. As with other viruses, this has been done with SARS-CoV-2 (101, 102). In one broadly-based screen (101), 26 genes of SARS-CoV-2 were cloned and overexpressed ectopically in HEK293T cells and assessed for their ability individually to antagonize IFNβ expression using an IFNβ promoter-driven reporter assay. Three nsp proteins (nsp1, 6, and 13) and one accessory protein (ORF6) displayed significant inhibitory activity toward IFNβ promoter activity (101). Likewise, nsp13 and ORF6 were independently identified as antagonists of the type I IFN response (103). Nsp6 and nsp13 then were shown to inhibit TBK1 and IRF3 activation, and ORF6 to inhibit nuclear translocation of transcription factor IRF3 (101) in the RLR-dependent IFN production signaling pathway (Fig. 2). To assess the effects of the SARS-CoV-2 proteins on JAK-STAT signaling, an ISRE promoter-driven reporter was utilized to measure inhibitory activity of the overexpressed viral proteins. With this assay a large number of SARS-CoV-2 proteins, nine, suppressed type I IFNα signaling: nsp 1, 6, 7, and 13; ORF 3a, 6, 7a, and 7b; and virion M protein. Subsequent examination showed that nsp6, nsp13, and ORF7b inhibited the phosphorylation of both STAT1 and STAT2; and, that ORF6 inhibits type I IFN signaling by impairing STAT1 nuclear translocation (101). Findings regarding the ability of some of the nsp and ORF proteins to impair type I IFN responses differed between studies (48, 101, 103). Results implicating the structural M protein as an antagonist of the RLR (TRAF3-TANK-TBK1) MAVS signaling pathway, for example, differ between viruses and studies when assessed by ectopic overexpression (101, 104, 105).

M from SARS-CoV behaved as an antagonist (104). M from SARS-CoV-2 also behaved as an antagonist of type I and III IFN signaling in one study (105) but not as an inhibitor of type I IFNβ signaling in another (101). The reason for the differences observed between studies is unclear (48, 101, 103, 104, 105). Possibly, when viral proteins are expressed individually using ectopic overexpression strategies, the protein expression level and the subsequent subcellular localizations and interactions with other proteins differ from that occurring in virus-infected cells (15, 16, 21). It will be important to verify findings obtained by ectopic overexpression of viral proteins individually with those obtained by infection with SARS-CoV-2 or engineered mutants thereof. The nucleocapsid protein N from SARS-CoV-2 also is described as an antagonist of RLR signaling and IFN expression by suppressing stress granule formation and thereby promoting replication (106). It is unknown whether the antagonistic activity shown by N is the consequence of overexpression of a protein that possesses RNA binding activity, as stress granule formation may be triggered by dsRNA and potentially suppressed by any RNA binding protein that sequesters dsRNA, as well as by the ISG p150 ADAR1, which destabilizes dsRNA structure (107, 108).

Virus replication and IFN antagonism by SARS-CoV and SARS-CoV-2 were also compared in cell culture by assessing the virus replication and viral sensitivity towards type I IFN treatment and cytokine induction using RT-PCR and promoter-reporter LUC plasmid expression assays, with a focus on ORF6 (64). Replication was higher for SARS-CoV in Vero E6 cells and for SARS-CoV-2 in Calu3 cells. SARS-CoV-2 was more sensitive to IFN treatment and less efficient in suppressing cytokine induction *via* IRF3 nuclear translocation. A reverse genetics approach was taken to compare the ORF6 antagonistic function between SARS-CoV-2 and SARS-CoV viruses. SARS-CoV-2 ORF6 expressed in the context of a fully replicating SARS-CoV virus backbone suppressed induction of *MX1*, but this suppression was less efficient than that by ORF6 from SARS-CoV (64). Mx is an ISG that typically shows very low basal expression and, hence, whose induction is exquisitely sensitive to IFN.

When SARS-CoV-2 proteins were compared to those of SARS-CoV and MERS-CoV for inhibitory activity in reporter assays, the nsp1, nsp6, and nsp13 proteins from all three coronaviruses comparably inhibited IFNβ promoter activity. By contrast, nsp1 and nsp6 of SARS-CoV and MERS-CoV were weaker inhibitors of IFNα signaling than those of SARS-CoV-2 (101). Assessment of viral gene products individually by the ectopic overexpression strategy would readily permit examination of mutated forms of the proteins compared to wild type for antagonistic activity. Study of the impairment of host IFN responses in virus-infected cells, including with engineered mutant forms of highly pathogenic coronaviruses (SARS-CoV, MERS-CoV, and SARS-CoV-2) requires appropriate biosafety containment and operating procedures. Studies of mutant viruses have been done though, for example, with MHV mutated to not express the nsp2 2′,5′-PDE enzyme that antagonizes the 2′,5′-oligo A-dependent RNase L response (109), or as illustrated by the reverse genetics approach using the SARS-CoV virus backbone for functional analysis of SARS-CoV-2 ORF6 protein in replicating virus described earlier herein (64).

### ORF6

The viral accessory protein ORF6 is consistently observed in studies to exert an anti-IFN activity. ORF6 from both SARS-CoV (110) and SARS-CoV-2 (48, 101) is sufficient to inhibit STAT nuclear translocation and, hence, type I IFN signaling. Mechanistically the ORF6 protein of SARS-CoV-2 localizes to the nuclear pore complex to block STAT1 and STAT2 nuclear import, thereby impairing IFN signaling and the STAT-dependent transcriptional activation of ISG expression (111). SARS-CoV-2 ORF6 directly interacts with Nup98 to inhibit docking of the cargo-receptor (karyopherin/importin) complex and disrupt nuclear import of the activated ISGF3 complex containing the STAT1 and STAT2 transcription factors.

### PLpro

The SARS-CoV-2-encoded papain-like protease PLpro (a modular domain within nsp3) antagonizes the action of IFN-induced gene ISG15 to impair the innate response and viral spread (112). Although the SARS-CoV-2 PLpro and the SARS-CoV PLpro share ∼83% sequence identity, the enzymes exhibit different host substrate preferences. SARS-CoV-2 PLpro preferentially cleaves the ubiquitin-like ISG15 protein, while SARS-CoV PLpro, by contrast, predominantly targets ubiquitin chains (112). Differences are also seen for the nsp5 chymotrypsin-like protease CLpro; nsp5 from SARS-CoV-2 had higher activity than the SARS-CoV ortholog for cleavage at multiple sites of NEMO, a key kinase in the RLR response pathway (113).

### PDE

MHV, like SARS-CoV-2, is a betacoronavirus. MHV encodes a viral phosphodiesterase (PDE) that antagonizes the OAS-RNase L antiviral response by degrading of the 2′-5- oligoadenylate produced by the dsRNA-activated OAS synthetases (109). Unlike MHV, SARS-CoV-2 virus does not encode a similar PDE enzyme.

## IFN treatment protects against SARS-CoV-2 virus in the mouse model

IFN λ protects against SARS-CoV-2 in different mouse models (114, 115). SARS-CoV-2 cannot infect wild-type mice because of inefficient interaction between the viral spike S protein and the mouse orthologue of the human receptor, ACE2 (7). Thus, mouse models for SARS-CoV-2 have been generated that display varying degrees of viral replication and clinical disease. These include human *ACE2* transgenic mice (115, 116) and a recombinant mouse-adapted (MA) SARS-CoV-2 virus, in which the interaction between the viral SARS-CoV-2 spike protein was engineered to bind the mouse ACE2 receptor (114). Hence, in the mouse-adapted SARS-CoV-2 MA model, the mouse ACE2 protein is used for entry into cells in order to initiate infection (114). SARS-CoV-2 MA replicates in the upper and lower airways of both young adult and aged BALB/c mice. SARS-CoV-2 MA infection caused more severe disease in aged mice and exhibited more clinically relevant phenotypes than those seen in transgenic mice that express human ACE2 (114). Both prophylactic and therapeutic administration of pegylated IFN λ1 (pegIFN λ1) significantly reduced weight loss, diminished SARS-CoV-2 MA replication in the lung, and protected mice from pulmonary dysfunction. Additionally, pegIFNλ1 treatment reduced the SARS-CoV-2 MA titer in the lungs of mice.

Nasally delivered murine IFN λ2 also protected hACE2 transgenic mice (expressing the human ACE2 receptor) against infection by SARS-CoV-2, as measured by reduced upper and lower respiratory tract infection and inflammation (115). IFN λ protected mice against infection with SARS-CoV-2 beta and omicron variants, whereas IFN λ receptor mutant (*IL28Rα−/−)* C57BL/6 mice sustained higher viral loads in the respiratory tract in the absence of a functional IFN λ receptor. IFN λ selectively induces antiviral ISG genes without causing excess inflammation. In the lung, IFN λ is produced mainly by lung epithelial cells *via* MAVS (RLR) and Myd88 (TLR) signaling pathways (115). Dysregulated type I IFN and inflammatory monocyte macrophage responses were previously shown to cause lethal pneumonia in SARS-CoV-infected mice (95). Using this SARS-CoV mouse model, robust viral replication occurs, accompanied by a delayed type I IFN response and inflammatory responses and lung immunopathology. Early type I IFN treatment improved immunopathology (95). Genetic knockout of the IFNAR receptor protected mice from lethal infection without affecting viral load.

## IFN system genetic determinants of human COVID-19 disease

In some cases of SARS-CoV-2 infection, critical hypoxemic pneumonia develops, but SARS-CoV-2 infection does not result in critical disease in all individuals. Why? Human genetic determinants of critical COVID-19 pneumonia have been identified in the IFN system (68, 117, 118). Inherited and or autoimmune deficiencies of the type I IFN response may account for ∼15%-20% of the cases of critical COVID-19 pneumonia in unvaccinated individuals (43). Recessive or dominant inborn errors of IFN immunity can underlie the COVID disease in unvaccinated adults. Inborn errors of IFN immunity also are seen in children (119, 120). Pre-activated antiviral innate immunity has been described in children as a factor in upper airways that controls early SARS-CoV-2 infection, with a higher basal expression of the viral RNA sensors MDA5 and RIG-I in cells from children than from adults (119).

### TLR3, TLR7, TBK1, IRF7, IFNAR1, and STAT2

Inborn errors of IFN system components (Fig. 2) identified from patients include those with TLR3 and TLR7 deficiencies that affect the production of type I IFNs by respiratory epithelial and plasmacytoid dendritic cells, and TBK1 and IRF7 deficiencies that also affect IFN production (68, 121). Additional deficiencies identified occur in IFN system genes for IFNAR, Tyk2, and STAT2 that affect JAK-STAT signaling and hence IFN action (67, 68, 117, 122). Plasmacytoid dendritic cells are the predominant IFN source following sensing of SARS-CoV-2 infection, and they restrict SARS-CoV-2 spread by a type I and type III IFN response, with responsiveness inversely correlating with the severity of disease (123). Types I and III IFNs appear to be potentially good markers to monitor COVID-19 pathophysiology (124).

### OAS

The human chr12q24.13 locus encodes three IFN-inducible 2′-5′-oligoadenylate synthetase enzymes (OAS1-3). OAS proteins, when activated by dsRNA, produce 2′,5′-oligoadenylate products that mediate dimerization and activation of the latent cellular RNase L, thereby triggering RNA degradation in infected cells (25, 30). A polymorphism in the human *OAS1* gene is associated with the clearance of SARS-CoV-2 virus (125). Genetic regulation of *OAS1* nonsense-mediated decay underlies the association with COVID-19 hospitalization (125). Only the OAS1 ISG isoform appears functionally critical for anti-SARS-CoV-2 activity, and the early control of SARS-CoV-2 replication through OAS1 appears to be an important determinant of COVID-19 disease severity (39, 125, 126). A common haplotype comprised of derived human risk alleles of two *OAS1* expression variants is associated with the risk of hospitalized COVID-19 in patients of European and African ancestries, compared to non-hospitalized patients (125).

## IFN system immunological determinants of human COVID-19 disease

Auto-antibodies directed against type I IFNs emerged as an important determinant of critical COVID-19 disease (117, 127). The prevalence in the general population of auto-antibodies neutralizing type I IFN α subspecies, but not IFN β, increases with age and is higher in men than women. It is estimated that as high as ∼15 % of the patients with critical COVID-19 pneumonia disease show significant auto-antibody titers against type I IFNs, whereas individuals with asymptomatic SARS-CoV-2 infection do not possess such titers (43, 128, 129, 130, 131). The action of type I IFNs also is impaired in individuals with auto-antibodies, as revealed by reduced ISG expression (132, 133). The risk of COVID-19 death is much greater and age-dependent for individuals with type I IFN autoantibodies (134), further suggesting the importance of a properly regulated IFN response for protection from critical COVID-19 disease. Based on auto-antibody profiles, type I IFNω appears to play an important role in nasal type I IFN immunity (132). The nasal IFN I/III signature correlated with nasopharyngeal viral load. However, a subset of critically ill COVID-19 patients showed low IFN I/III despite high nasal viral loads that correlated with the presence of auto-antibodies against type I IFN (132).

## Therapeutic use of IFN in patients with SARS-CoV-2 infection

The potential for using IFN types I or III as a treatment strategy for SARS-CoV-2 infection and COVID-19 disease has been reviewed against the background experience of the benefit of IFN therapy in the treatment of SARS-CoV and MERS-CoV, two related coronavirus infections (135).

### Type III IFN λ

Some studies of *IFN λ* (135, 136, 137), including a large phase 3 randomized placebo-controlled trial, showed that patients with COVID-19 treated with the recombinant, pegIFN λ had improved SARS-CoV-2 viral clearance (136). Among patients with a high SARS-CoV-2 viral load at baseline, those who received pegIFN λ had lower viral loads by day 7 than those individuals who received a saline placebo, with the incidence of adverse events similar in the two groups (136). An earlier phase 2 study of the effect of pegIFN λ also indicated that pegIFN λ treatment accelerated viral decline in outpatients with COVID-19, increasing the proportion of patients with viral clearance by day 7, particularly in those with a high baseline viral load (138). By contrast, a different study indicated little promise of IFN λ treatment; a single dose of subcutaneous pegIFN λ neither shortened the duration of SARS-CoV-2 viral shedding nor improved symptoms in outpatients with uncomplicated COVID-19 (139).

### Type I IFNs α and β

Trials in patients with COVID-19 have reported that both subcutaneous and inhaled type I IFN administration may reduce the duration of viral shedding and disease symptoms. An uncontrolled exploratory study of the effect of IFNα2b treatment of a cohort of confirmed COVID-19 cases in Wuhan, China, showed that the α IFN with or without arbidol significantly reduced the duration of detectable SARS-CoV-2 virus in the upper respiratory tract and, in parallel, reduced duration of elevated blood levels for the inflammatory marker IL-6 (140).

The safety and efficacy of inhaled nebulized IFNβ1a (SNG001) for treatment of SARS-CoV-2 infection in a randomized, double-blind, placebo-controlled, phase 2 trial showed that patients who received SNG001 had greater odds of improvement and recovered more rapidly from SARS-CoV-2 infection than patients who received placebo (141). The triple combination of IFNβ1b, lopinavir–ritonavir, and ribavirin in the treatment of patients admitted to hospital with COVID-19 in a randomized, phase 2 trial indicated that the triple antiviral therapy was both safe and superior to lopinavir–ritonavir alone in alleviating symptoms and shortening the duration of viral shedding and hospital stay in patients with mild to moderate COVID-19 (142). However, the efficacy of type I IFN for the treatment of COVID-19, like type III IFN, is not consistently seen in all studies. For example, in a double-blinded, placebo-controlled trial the combination of IFNβ plus remdesivir showed no clinical benefit when compared to remdesivir alone, suggesting no clinical benefits of the IFNβ therapy in patients with COVID-19 (143). The World Health Organization Solidarity trial also did not show a benefit for IFNβ (144). The presence of auto-antibodies against type I IFNs seen in a subset of patients with critical COVID-19 disease, as discussed earlier, may limit the value of type I IFN as a therapeutic, particularly in patients displaying especially high anti-IFN antibody titers. Evidence suggests that defects in the type I IFN response are of prime importance in determining the severity of COVID disease (99, 145). While IFN importantly can control SARS-CoV-2 viral replication at early times of infection, it may also exacerbate inflammatory disease at later times (19, 145, 146, 147). Furthermore, COVID-19 patient morbidity has been reported to correlate with high expression of type I and III IFNs in the lung. These IFNs can disrupt the lung epithelial barrier upon viral infection (148, 149).

## Long COVID and the IFN response

Long COVID or chronic COVID syndrome refers to the long-term effects of COVID-19 disease that occur with symptoms affecting multiple organ systems that persist following acute COVID-19 disease (150, 151, 152, 153, 154). Among the factors that appear to play a role in Long COVID is dysregulation of the IFN response (150, 155). Altered levels of proinflammatory cytokines IL-1β, IL-6, and TNFα, in addition to type II IFNγ and also types I and III IFNs, are seen in individuals recovered from COVID-19 compared to those with severe disease (47, 52, 150, 156, 157). SARS-CoV-2 persistence also has been proposed, and viral persistence may be affected by IFN (131, 158).

Analysis of SARS-CoV-2 infection and persistence in the human body at autopsy also suggests that the SARS-CoV-2 virus can result in systemic infection and persist (152). The virus was found widely distributed in patients who died of COVID-19; SARS-CoV-2 virus replication was present in multiple respiratory and non-respiratory tissues, including the brain. Persistent viral RNA was detected at multiple sites throughout the body, though little evidence was found for direct viral cytopathic effect or inflammation (152).

Genetic disorders within the type I IFN system in plasmacytoid dendritic cells (pDC) and respiratory epithelial cells that lead to dysregulation of the IFN production response also are associated with severe COVID disease (68, 121, 146, 159). IFN treatment interestingly also may contribute to cell death and lethality during betacoronavirus infection (160). An intriguing possibility is that this cytotoxicity may involve the IFN-inducible p150 adenosine deaminase ADAR1 and the ZBP1 protein. Both proteins are IFN inducible and contain the Zα domain that recognizes Z-RNA, an alternative left-handed double-helix RNA structure (81). ZBP1 is the only cellular protein in mammals other than p150 ADAR1 that is known to possess Zα. ADAR1 averts fatal type I IFN induction by ZBP1 (161). ZBP1-dependent inflammatory cell death, PANoptosis, happens in mouse and human macrophages and in the lungs of mice infected with SARS-CoV-2 and MHV. Expression of ZBP1 was increased in COVID-19 patients who succumbed to the disease compared to those that recovered (77). ZBP1 might contribute to type I interferonopathies caused by *ADAR1* mutations, and inhibition of ZBP1 may improve the efficacy of IFN therapy (38, 77).

## Conclusion

The emergence of pathogenic coronaviruses has happened three times during the past ∼ 20 years: 2002 with SARS-CoV; 2012 with MERS-CoV; and, most recently with the 2019 SARS-CoV-2 virus and the COVID-19 pandemic. The three plus years of COVID-19 caused by SARS-CoV-2 brought a multitude of challenges and opportunities, for science and for society. Rapid diagnostic assays, effective vaccines, and antiviral therapies against SARS-CoV-2 were rapidly developed and together are continuing to contribute to our emergence from the COVID-19 pandemic. The potential, however, for the future emergence of an even more fit SARS-CoV-2 variant or an altogether new coronavirus remains a possibility, given the likely zoonotic nature of SARS-CoV-2 and the bat reservoir of many additional coronaviruses. Hence, it is important to understand in molecular terms the host responses to SARS-CoV-2 infection, including the IFN response.

Interferon is our first response to infection. Dysregulation of type I IFN responses, however, is seen in SARS-CoV-2 infection and COVID-19, where IFN production often is blunted and expression of pro-inflammatory cytokines enhanced. The importance of a balanced type I IFN response is illustrated by the severe disease status that may develop in patients with genetic or immunological defects in the type I IFN response pathways for IFN production and IFN action. While considerable progress has been made toward understanding both the molecular details of the SARS-CoV-2 multiplication cycle and the pathways by which IFNs are induced, much remains to be learned with regard to the biochemical mechanisms by which ISGs, likely multiple ISGs acting in combination, function to inhibit SARS-CoV-2 replication. The SARS-CoV-2 virus is IFN-sensitive. Both type I IFN α subspecies and β, and type III IFN λ subspecies, hold potential as therapeutics. However, the conditions for their optimal and effective use against pathogenic coronavirus infection are just being learned. A number of SARS-CoV-2 gene products, including both nsps and accessory ORF proteins possess inhibitory activities that suppress the IFN response. Whether it will be possible to use knowledge, for example, from the protein interaction map for SARS-CoV-2 viral and cellular proteins, to develop effective drugs that enhance IFN antiviral activity is currently unknown. IFN protects against SARS-CoV-2 infection. Genetic and immunologic determinants of the IFN response have been identified that affect COVID disease severity and outcome, further illustrating the importance of understanding and optimizing the IFN response, both as an endogenously induced first-line defense and potentially as an exogenously administered therapeutic.

## Conflict of interest

The author declares that he has no conflicts of interest with the contents of this article.
